# Sulfur-Doped Graphdiyne as a High-Capacity Anode Material for Lithium-Ion Batteries

**DOI:** 10.3390/nano11051161

**Published:** 2021-04-29

**Authors:** Fanan Kong, Yong Yue, Qingyin Li, Shijie Ren

**Affiliations:** State Key Laboratory of Polymer Materials Engineering, College of Polymer Science and Engineering, Sichuan University, Chengdu 610065, China; xlfc12138@stu.scu.edu.cn (F.K.); 2018223090115@stu.scu.edu.cn (Y.Y.); qingyinli@scu.edu.cn (Q.L.)

**Keywords:** sulfur-doped graphdiyne, bottom-up synthesis, anode material, lithium-ion batteries

## Abstract

Heteroatom doping is regarded as a promising approach to enhance the electrochemical performance of carbon materials, while the poor controllability of heteroatoms remains the main challenge. In this context, sulfur-doped graphdiyne (S-GDY) was successfully synthesized on the surface of copper foil using a sulfur-containing multi-acetylene monomer to form a uniform film. The S-GDY film possesses a porous structure and abundant sulfur atoms decorated homogeneously in the carbon skeleton, which facilitate the fast diffusion and storage of lithium ions. The lithium-ion batteries (LIBs) fabricated with S-GDY as anode exhibit excellent performance, including the high specific capacity of 920 mA h g^−1^ and superior rate performances. The LIBs also show long-term cycling stability under the high current density. This result could potentially provide a modular design principle for the construction of high-performance anode materials for lithium-ion batteries.

## 1. Introduction

As one of the most promising energy storage devices, rechargeable lithium-ion batteries (LIBs) have drawn much attention because of their large energy density, high working voltage, and long cycling life [1,2]. Electrode materials are the core components of LIBs and largely determine their ultimate performance, and the research on anode materials has received extensive attention [3,4,5]. Carbon materials, including graphite and relatively newly developed carbon nanotubes and graphene, are the most used anode materials for LIBs [6,7,8,9]. However, their application in LIBs is restricted by limited lithium storage capacity, low-rate capability, poor capacity retention, etc. [10]. Thus, it is still of great interest to develop novel carbon materials for LIBs application.

Recently, a newly artificial carbon allotrope, graphdiyne (GDY), which is constructed by sp^2^ and sp carbon atoms, has been developed [11]. Due to its two-dimensional all-carbon structure and intrinsic nanoporosity, GDY and its derivatives exhibit promising electronic, optical, and mechanical properties and thus have been extensively studied as active materials in many different applications [12,13,14]. In the energy storage area, GDY-based materials exhibit a great potential to be used as anode materials for LIBs with large capacity, high output power, and excellent stability [15]. To improve the electrochemical performance of GDY further, heteroatoms such as nitrogen (N) [16,17], sulfur (S) [18], chlorine (Cl) [19], hydrogen (H) [20], boron (B) [21] and fluorine (F) [22] have been doped into GDY to improve its conductivity and wettability. Thermal annealing of GDYs at high temperatures with the presence of heteroatom-containing small molecules is a convenient top-down method for the preparation of heteroatom-doped GDYs (HD-GDYs). However, this method suffers from some drawbacks, including uncertainty of doping sites and uncontrollability of doping amounts. Another method to prepare HD-GDYs is the direct polymerization of heteroatom-containing multi-acetylene monomers. This bottom-up method could essentially control the content and location of the heteroatoms. Among these HD-GDYs, nitrogen-doped GDYs have been extensively studied and used as highly promising candidates for the fabrication of advanced energy storage devices [17,23,24].

Compared with nitrogen, sulfur has a larger atom size and a lower electronegativity. Doping sulfur into two-dimensional carbon materials could expand the interlayer distance, create active sites, and significantly boost the electronic properties of carbonaceous materials [25,26]. Recently, some studies have revealed that sulfur-doped GDYs (S-GDYs) prepared by the top-down method displayed improved electrochemical properties in LIBs, including high-rate performance and excellent stability [27,28]. Meanwhile, bottom-up synthesis of sulfur-containing conjugated polymers, especially those based on polythiophene, has been studied for energy storage applications due to their high intrinsic conductivity and abundant electroactive sites [29]. For instance, Zhang et al. reported the structural design of thiophene-containing conjugated microporous polymers as high-performance anode materials for LIBs [30]. Recently, Wang et al. prepared thiophdiyne films as artificial protective layers for aluminum foil in dual-ion batteries, resulting in improved cycling stability [31]. However, there has been no report on the application of thiophene-based S-GDYs as the anode materials for LIBs. In this work, we designed and prepared sulfur-doped graphdiyne (S-GDY) with extended π-conjugated carbon skeletons comprised of butadiyne linkages and thiophene rings through a bottom-up synthetic strategy. The obtained S-GDY exhibits enhanced electrochemical performance due to the synergistic effect of sulfur doping and porous structure.

## 2. Materials and Methods

### 2.1. Materials

Trimethylsilyl acetylene, 2,3,4,5-tetrabromothiophene, potassium fluoride, 1,3,5-tribromobenzene, bis(triphenylphosphine)palladium (II) dichloride, copper (I) iodide, trimethylamine, pyridine, and tetrahydrofuran were all purchased from Adamas. Tetrahydrofuran was dried by distillation and other chemicals were used as received without any further purification.

### 2.2. Experimental Section

The monomer 2,3,4,5-tetraethynylthiophene (M1) was obtained following the synthetic route shown in Figure S1.

#### 2.2.1. Synthesis of 2,3,4,5-tetrakis[(trimethylsilyl)ethynyl]-thiophene (2)

Under Ar atmosphere, trimethylsilyl acetylene (1.47 g, 15 mmol), 2,3,4,5-tetrabromothiophene (1 g, 2.5 mmol), bis(triphenylphosphine)palladium (II) dichloride (53 mg, 0.075 mmol), and copper (I) iodide (14 mg, 0.075 mmol) were dissolved in the triethylamine (60 mL). The reaction mixture was heated to 80 °C and stirred for 24 h. Then, water (200 mL) was added and the water phase was extracted with dichloromethane and dried over Na_2_SO_4_. The solvent was evaporated and the residue was chromatographed on silica gel with n-hexane to give 2,3,4,5-tetrakis[(trimethylsilyl)ethynyl]-thiophene as white solid (480 mg, 41%). ^1^H NMR (400 MHz, CDCl_3_): δ (ppm) 0.27. ^13^C NMR (101 MHz, CDCl_3_): δ (ppm) 121.17, 119.49, 105.62, 95.02, −0.36. Anal. Calcd for C_24_H_36_SSi_4_: C, 61.47; H, 7.74; S, 6.48. Found: C, 61.77; H, 7.53; S, 6.65.

#### 2.2.2. Synthesis of 2,3,4,5-tetraethynylthiophene (M1)

Under Ar atmosphere, 2,3,4,5-tetrakis[(trimethylsilyl)ethynyl]-thiophene (50 mg, 0.11 mmol) and potassium fluoride (95.7 mg, 1.65 mmol) were in the mixture of tetrahydrofuran (10 mL), methanol (10 mL). The reaction was stirred at room temperature for 2 h. After 200 mL of water was added, the reaction mixture was extracted with ether and dried over Na_2_SO_4_. Since 2,3,4,5-tetraethynylthiophene is modestly stable in solvent at room temperature, the solvent was evaporated, and then pyridine (50 mL) was added under nitrogen atmosphere at room temperature. The solution was used for the next step. ^1^H NMR (400 MHz, CDCl3): δ (ppm) 3.58 (s, 4H). ^13^C NMR (101 MHz, CDCl_3_): δ (ppm) 120.98, 119.93, 86.72, 74.68.

#### 2.2.3. Preparation of S-GDY Film

Copper foil (area: 2 × 2 cm^2^, thickness: 11 ± 2 μm) was washed with 4 M hydrochloric acid (HCl), water, ethanol, and acetone, respectively, and dried under Ar atmosphere. Several (10) pieces of copper foil (area: 2 × 2 cm^2^) and pyridine (50 mL) were added to a three-neck flask (250 mL). The mixture was heated at 60 °C under Ar for 1 h. The above solution with 2,3,4,5-tetraethynylthiophene and pyridine was transferred to an Ar-protected constant addition funnel and added dropwise into the mixture. After the addition of 2,3,4,5-tetraethynylthiophene, the reaction mixture was maintained at 60 °C for 3 days. Upon completion of the reaction, the precipitation was filtered and washed with distilled water, ethanol, acetone, CHCl_3_, and methanol. After calcined at 400 °C for 2 h, a black film was obtained on the copper foil and the thickness of S-GDY film was around 1.46 µm.

### 2.3. Structure Characterizations

Fourier transform infrared (FT–IR) spectra were recorded on a Nicolet 560 Fourier transform IR spectrometer (Thermo Scientific, Waltham, MA, USA). The XRD patterns were recorded using a Philips X’Pert PRO MPD (PANalytical B.V., Malvern, Holland). The Raman spectra were recorded on a LabRAM HR Raman Microscope (HORIBA Scientific, Paris, France) with an excitation length of 633 nm and a laser power of 0.5 mW. Morphology details were examined using scanning electron microscopy (SEM, JEOL JSM-5900LV, JEOL Ltd., Tokyo, Japan), transmission electron microscopy (TEM, FEI Tecnai G2 F20 S-TWIN, FEI Company, Hillsboro, OR, USA). The STEM and elemental mapping images were obtained from FEI Titan G2 60-300 (AC-TEM, FEI Company, Hillsboro, OR, USA). X-ray photoelectron spectroscopy (XPS) measurement was carried out on a Kratos ASAM 800 analyzer (Kratos Analytical Ltd., Manchester, UK), performing at 12 kV and 15 mA with a monochromatic Al Kα source (hν = 1486.6 eV). The nitrogen and adsorption–desorption measurements were performed on a BELSORB Max (MicrotracBEL Corp., Tokyo, Japan) sorption analyzer. The surface areas were calculated using the BET model in the pressure range *P*/*P_0_* from 0.05 to 0.3. The total pore volume was determined at a relative pressure of 0.99. The pore size distribution was analyzed from the gas adsorption data using an NLDFT method with a slit pore model.

### 2.4. Electrochemical Measurements

We used a typical Li-ion battery to estimate the electrochemical performances of S-GDY. Electrochemical experiments were performed using CR2032-type coin cells. The S-GDY films grown on the copper foil were cut into a round shape (diameter = 12 mm) and directly used as working electrodes without the addition of any binders. The current densities and capacities were calculated based on the active material of S-GDY with a low mass loading of about 0.24 mg/cm^2^. A pure Li foil was used as the counter electrode. The counter electrode was separated from the working electrode by a Celgard 2500 polymeric separator. The electrolyte consists of a solution of 1 M LiPF_6_ in ethylene carbonate (EC) and dimethyl carbonate (DMC) (1:1, *v*/*v*). We assembled the cells in an argon-filled glove box. The assembled half cells were cycled between 0.005 and 3 V using a LAND battery testing system (Wuhan, China). Cyclic voltammetry (CV) was carried out using a CHI760E electrochemical workstation (Shanghai, China) between 5 mV and 3 V vs. Li/Li^+^ at a scan rate of 1 mV/s. Electrochemical impedance spectroscopy (EIS) was performed on a CHI760E electrochemical workstation by applying an AC voltage of 5 mV amplitude at room temperature.

## 3. Results and Discussion

**Figure 1:**
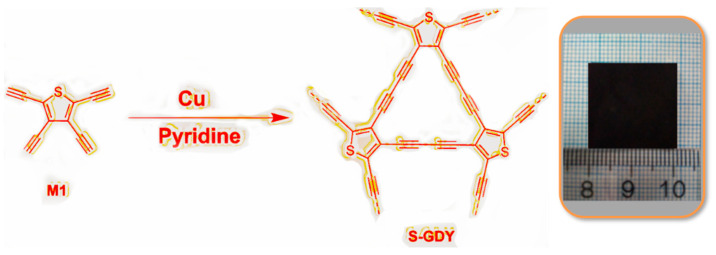
The preparation route and the photograph of S-GDY film on the copper foil.

Herein, S-GDY with extended π-conjugated carbon skeletons comprised of butadiyne linkages and thiophene rings was prepared by a bottom-up synthetic strategy. The monomer 2,3,4,5-tetraethynylthiophene (M1) was synthesized by a two-step synthesis. The synthetic route and related NMR spectra are described in the Supplementary Materials (Figures S1–S5, ESI†). Then, S-GDY was synthesized through an in situ cross-coupling reaction of the monomer M1 on copper foil in pyridine as a uniform black film (Figure 1). In the process of forming S-GDY films, the polymer particles are obtained by the catalysis of Cu (II) ion arising from the surface of the Cu foil in alkaline solution and then deposited on the copper foil to form a film [32]. For the convenience of characterizations, the S-GDY film could be collected by the dissolution of the copper substrate in a saturated FeCl_3_ solution.

The chemical structure of the sulfur-doped graphdiyne was confirmed by Fourier transform infrared spectroscopy (FT–IR) and Raman spectroscopy. The peaks at 1645 cm^−1^ and 1411 cm^−1^ in Figure S6 can be assigned to the skeletal vibrations of the thiophene ring [30]. Meanwhile, a weak peak at around 2200 cm^−1^ corresponds to the typical C≡C stretching vibration. Unlike Raman spectra of common carbon materials that exhibit obvious vibration peaks at the D band and G band, corresponding to the structural defects and highly ordered graphite carbon, respectively, the Raman spectrum of S-GDY presents a single G band vibration peak at 1533 cm^−1^ (Figure S7, ESI†), indicating a high regularity of the S-GDY film on the surface of copper foil. The weak peaks at 2142 cm^−1^ and 2285 cm^−1^ can be attributed to the stretching vibration of conjugated butadiyne linkage (-C≡C-C≡C-) [33]. In addition, a broad 2D peak at 2800 cm^−1^ indicates that a multilayered structure exists [34]. Powder XRD patterns of S-GDY film show a broad peak at approximately 24° (Figure S8, ESI†), indicating an overall amorphous structure, corresponding to a random accumulation of S-GDY at the macroscopic scale.

**Figure 2:**
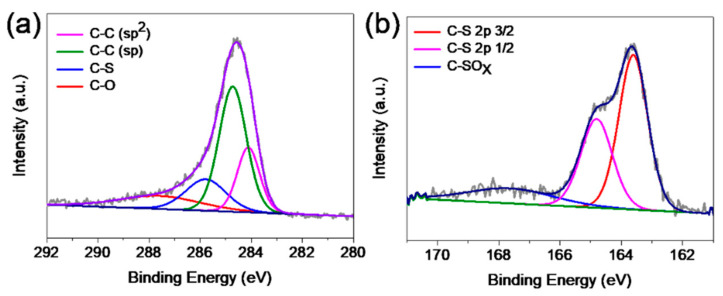
XPS spectra results: (**a**) C 1s binding energy profiles and (**b**) S 2p binding energy profiles.

The quantity and species of elements in S-GDY were further investigated by X-ray photoelectron spectroscopy (XPS) analysis. XPS survey spectra of S-GDY show the main elements of O, C, and S (Figure S9, ESI†). The O 1s peak located at about 532.0 eV could be mostly assigned to the adsorbed atmospheric oxygen-containing molecules and the oxidation of the acetylene group in the margin of the two-dimensional molecular plane [19,35]. The S 2p peak is located at about 163.0 eV and the content ratio of the sulfur element is 7.48%, which is close to the theoretical value (7.7% in atomic number ratio). C 1s peak of S-GDY can be deconvoluted into four subpeaks, corresponding to the C=C (sp^2^) at a binding energy of 284.2 eV, C≡C (sp) at 284.7 eV, C-S at 285.8 eV, and C-O at 287.6 eV (Figure 2a) [36]. The high-resolution XPS spectra of S 2p (Figure 2b) reveal the presence of three kinds of S configurations. The two evident peaks at 163.6 and 164.8 eV belong to S atoms connected with carbon (-C-S-C-), which are in agreement with the reported 2p_3/2_ and 2p_1/2_ positions of thiophene S owing to their spin–orbit coupling [36,37]. The weak and broad peak at 169.1 eV is attributed to oxidized S (C-SO_x_-C) [38]. These results confirm the successful doping of sulfur atoms into graphdiyne skeleton, which could provide more heteroatom defects and active sites to improve Li storage ability.

**Figure 3:**
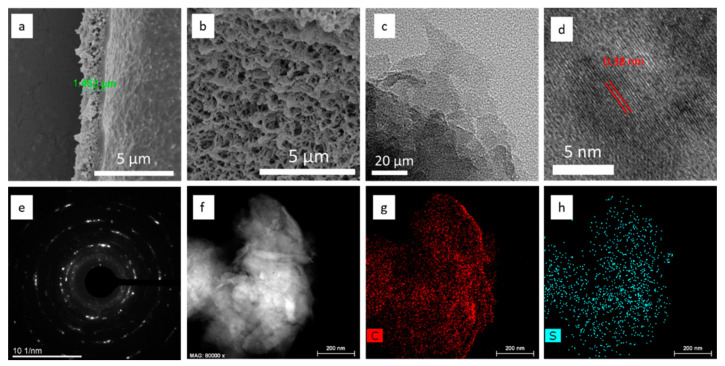
The morphology of S-GDY film: (**a**) the cross-section SEM image of the S-GDY film, (**b**) the top-view SEM image of the S-GDY film, (**c**) TEM image of the S-GDY, (**d**) HRTEM image of the S-GDY, (**e**) SAED pattern of the S-GDY, and (**f**) scanning transmission electron microscopy image and elemental mapping of (**g**) C-K and (**h**) S-K.

The morphology of the S-GDY film was investigated by scanning electron microscopy (SEM) and transmission electron microscope (TEM). The cross-section SEM image shows that the film is continuous and its thickness is approximately 1.46 μm (Figure 3a). Furthermore, the morphology of the side close to the copper substrate is more compact than that on the opposite side due to the template effect of the Cu foil. The top-view image shows that the S-GDY film has a rough surface with a large number of porous structures formed by the accumulation of small particles (Figure 3b), which would facilitate the movement and storage of electrolyte ions in LIBs application. TEM image (Figure 3c) shows the layer-by-layer stacking structure of S-GDY. High-resolution TEM (HRTEM) (Figure 3d) shows the interlayer spacing of S-GDY is 0.38 nm, which is larger than that of graphene (0.23 nm) [39] and graphdiyne (0.365 nm) [15]. Selected-area electron diffraction (SAED) patterns (Figure 3e) reveal high crystallinity of S-GDY in certain areas. Elemental mapping images of S-GDY show the homogeneous distribution of carbon and sulfur atoms in its skeleton structure (Figure 3f–h). The porous properties and corresponding specific surface area of the polymer were measured by nitrogen adsorption and desorption experiment. As shown in Figure S10a, the specific surface area of the polymer calculated from the BET algorithm is 35 m^2^ g^−1^, while the pore size distribution (PSD) curves in Figure S10b indicate that the pore size is mainly distributed in the mesoporous region.

**Figure 4:**
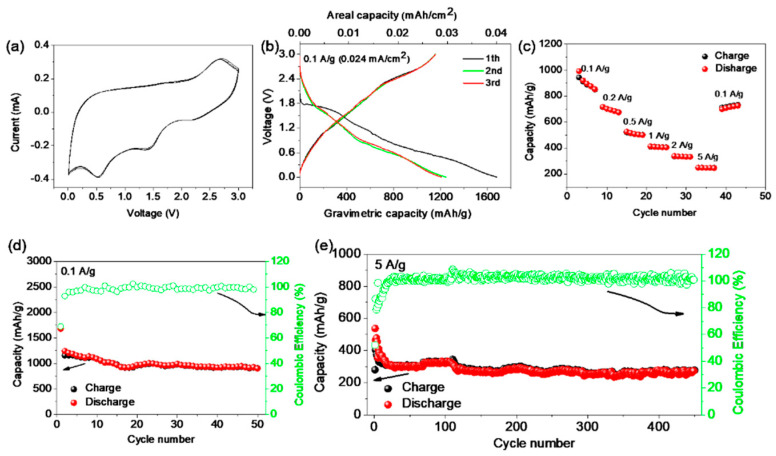
The electrochemical performances of S-GDY electrodes in Li metal half-cell format: (**a**) the first three cyclic voltammetry (CV) curves at a scan rate of 1 mV/s, (**b**) galvanostatic charge–discharge profiles at a current density of 0.1 A/g (0.024 mA cm^−2^), recorded between 5 mV and 3 V, (**c**) the rate performance, (**d**) the cycle performance at the current density of 0.1 A g^−1^, and (**e**) the cycle performance at the current density of 5 A g^−1^.

The electrochemical performances of S-GDY were evaluated by assembling S-GDY/LiPF_6_ electrolyte/Li metal half cells and testing in the potential range of 0.005–3.0 V vs. Li/Li^+^. The electrode is prepared by cutting S-GDY films grown on the copper foil into pieces without any addition of polymer binder and conductive agent. The first three cyclic voltammetry (CV) curves shown in Figure 4a were measured at a scan rate of 1 mV/s. It can be recognized that a cathodic peak is found at around 0.5 V, which represents the formation of a new chemical structure [22]. Another two redox peaks at 1.4 and 2.6 V correspond to the reversible redox reaction of the thiophene unit [30], suggesting that S-GDY exhibits electrochemical activity and stability. The galvanostatic charge–discharge profiles of the initial three cycles of the half cells at the current density of 0.1 A g^−1^ (0.024 mA cm^−2^) are shown in Figure 4b. At the first cycle, a large specific capacity of 1678 mA h g^−1^ (0.04 mA h cm^−2^) and high Coulombic efficiency (CE) of 68.8% are obtained from this electrode, indicating that doping S atoms could improve the Li adsorption ability and thus lead to a large capacity [40].

Rate performances of the S-GDY electrode from 0.1 to 5 A g^−1^ are shown in Figure 4c. The reversible gravimetric capacity of the S-GDY retains to be 248 mA h g^−1^, while the current density is increased to 5 A g^−1^, demonstrating that S-GDY prepared by this “bottom-up” method exhibits excellent rate capacities. In addition, long-term cycling performances of these electrodes are also measured. At a current density of 0.1 A g^−1^, the reversible capacity can reach 920 mA h g^−1^ after 50 cycles and the Coulombic efficiency on average is nearly 99% (Figure 4d), which is comparable to the state-of-the-art reported carbon-based materials (Table S1, ESI†). More intriguingly, even under higher current densities of 0.5 A g^−1^, 1 A g^−1^, S-GDY-based electrodes also display decent capacities of 675 mA h g^−1^ and 423 mA h g^−1^, respectively, after long cycles (Figures S11 and S12, ESI†). The specific capacity of S-GDY remains to be 275 mA h g^−1^ after 500 cycles under a current density of 5 A g^−1^, as shown in Figure 4e.

Electrochemical impedance spectroscopy (EIS) was employed to investigate the interfacial charge transfer and Li^+^ diffusion process. Nyquist plots and corresponding equivalent circuits of the S-GDY electrode before and after cycling at 0.5 A g^−1^ are shown in Figure S13. The semicircle in the Nyquist plot corresponds to the charge transfer process of electrodes before cycling, while the semicircle after 120 cycles is ascribed to combination resistance of solid electrolyte interphase (SEI) films and charge transfer process. The fitting kinetic parameters of the electrodes are listed in Table 1. It can be observed that the S-GDY electrode shows a much lower charge-transfer resistance (R_ct_) after cycling, indicating enhanced charge transportation ability and a higher Li^+^ diffusion rate during cycling [19]. In addition, the morphology of the S-GDY electrode after cycling was investigated and the results in Figure S14 reveal the formation of an SEI layer. All these results indicate that S-GDY is a promising anode material for LIBs probably due to the synergistic effect of sulfur doping and porous structure. The introduction of S atoms could provide more heteroatom defects and electrochemically active sites, and the porous structure facilitates the adsorption–desorption and diffusion of Li ions.

## 4. Conclusions

In conclusion, sulfur-doped graphdiyne (S-GDY), a distinctive two-dimensional heteroatom doped carbon material, was designed and prepared by a “bottom-up” method on the surface of copper foil using a sulfur-containing multi-acetylene monomer. The obtained S-GDY possesses abundant sulfur atoms decorated homogeneously in the carbon skeleton and exhibits enhanced electrochemical performance due to the synergistic effect of sulfur doping and porous structure. The S-GDY film proves to be a promising anode material with a highly reversible specific capacity up to 920 mA h g^−1^ at a current density of 0.1 A g^−1^ and excellent rate performances. The specific capacity of S-GDY can still retain 275 mA h g^−1^ after 500 cycles under a high current density of 5 A g^−1^. Overall, this “bottom-up” method could potentially provide a rational design principle for the construction of heteroatom-doped carbon materials to be used as high-performance anode materials for lithium-ion batteries.

## Figures and Tables

**Table 1:** Kinetic parameters of the S-GDY electrode before and after 120 cycles at the current density of 0.5 A g^−1^.

S-GDY	Before Cycles	After 120 Cycles
R_e_ (Ω)	5.7	4.5
R_s_ (Ω)	-	4.4
R_ct_ (Ω)	236.9	5.9

## Supplementary Materials

The following are available online at https://www.mdpi.com/article/10.3390/nano11051161/s1, Figure S1: Synthesis of 2,3,4,5-tetrakis[(trimethylsilyl)ethynyl]-thiophene (2) and 2,3,4,5-tetraethynyl thiophene (M1); Figure S2: ^1^H NMR spectrum of 2,3,4,5-tetrakis[(trimethylsilyl)ethynyl]-thiophene (2); Figure S3: ^13^C NMR spectrum of 2,3,4,5-tetrakis[(trimethylsilyl)ethynyl]-thiophene (2); Figure S4: ^1^H NMR spectrum of 2,3,4,5-tetraethynyl thiophene (M1); Figure S5: ^13^C NMR spectrum of 2,3,4,5-tetraethynyl thiophene (M1); Figure S6: FT–IR spectrum of S-GDY; Figure S7: Raman spectrum of S-GDY; Figure S8: Power XRD pattern of S-GDY; Figure S9: XPS survey spectrum of S-GDY; Figure S10: (a) Nitrogen adsorption-desorption isotherms of S-GDY, (b) pore size distribution profile from NLDFT calculation. Figure S11: Long-term capacity retention at a current density of 0.5 A g^−1^; Figure S12: Long-term capacity retention at a current density of 1 A g^−1^; Figure S13: (a) Nyquist plots of the S-GDY electrode before and after 120 cycles at 0.5 A g^−1^. The inset shows a S-GDY electrode after cycling alone in the enlarged part of the high-frequency region. (b) Equivalent circuits of the S-GDY electrode. In the equivalent circuit, R_e_ represents the electrolyte resistance; R_s_ is the equivalent resistance of the SEI layer formed on the electrode; R_ct_ is the charge-transfer resistance; Z_w_ is the Warburg impedance; CPE represents the corresponding double-layer capacitance. Figure S14: The SEM images of the S-GDY electrode after 120 cycles at 0.5 A g^−1^; Table S1: Comparison of the performance of previously reported carbon-based materials with S-GDY.
