# Supplementary material for: Sulfur-Doped Graphdiyne as a High-Capacity Anode Material for Lithium-Ion Batteries
Source: Nanomaterials (Basel). 2021 Apr 29;11(5):1161. doi: 10.3390/nano11051161 (PMC8145426; doi:10.3390/nano11051161)
Supplement: Supplementary file 1 [file nanomaterials-11-01161-s001.zip › nanomaterials-1175633-supplementary.pdf]

Supplementary Materials

Sulfur-Doped Graphdiyne as a High-Capacity Anode Material for Lithium-Ion Batteries

Fanan Kong, Yong Yue, Qingyin Li, and Shijie Ren *

State Key Laboratory of Polymer Materials Engineering, College of Polymer Science and Engineering, Sichuan University, Chengdu 610065, China

* Correspondence: rensj@scu.edu.cn

Contents

Section 1. NMR spectrum of the monomers

Section 2. Results of structure characterizations

Section 3. Results of porosity measurements

Section 4. Results of Electrochemical performances

Section 1. NMR spectrum of the monomers

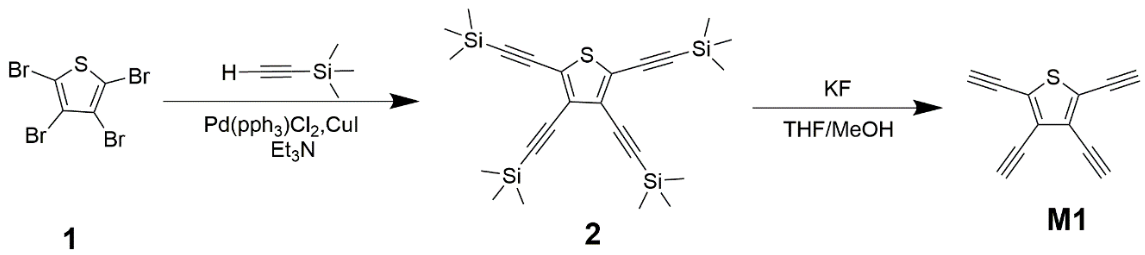

Figure S1. Synthesis of 2,3,4,5-tetrakis[(trimethylsilyl)ethynyl]-thiophene (**2**) and 2,3,4,5-tetraethynyl thiophene (**M1**).

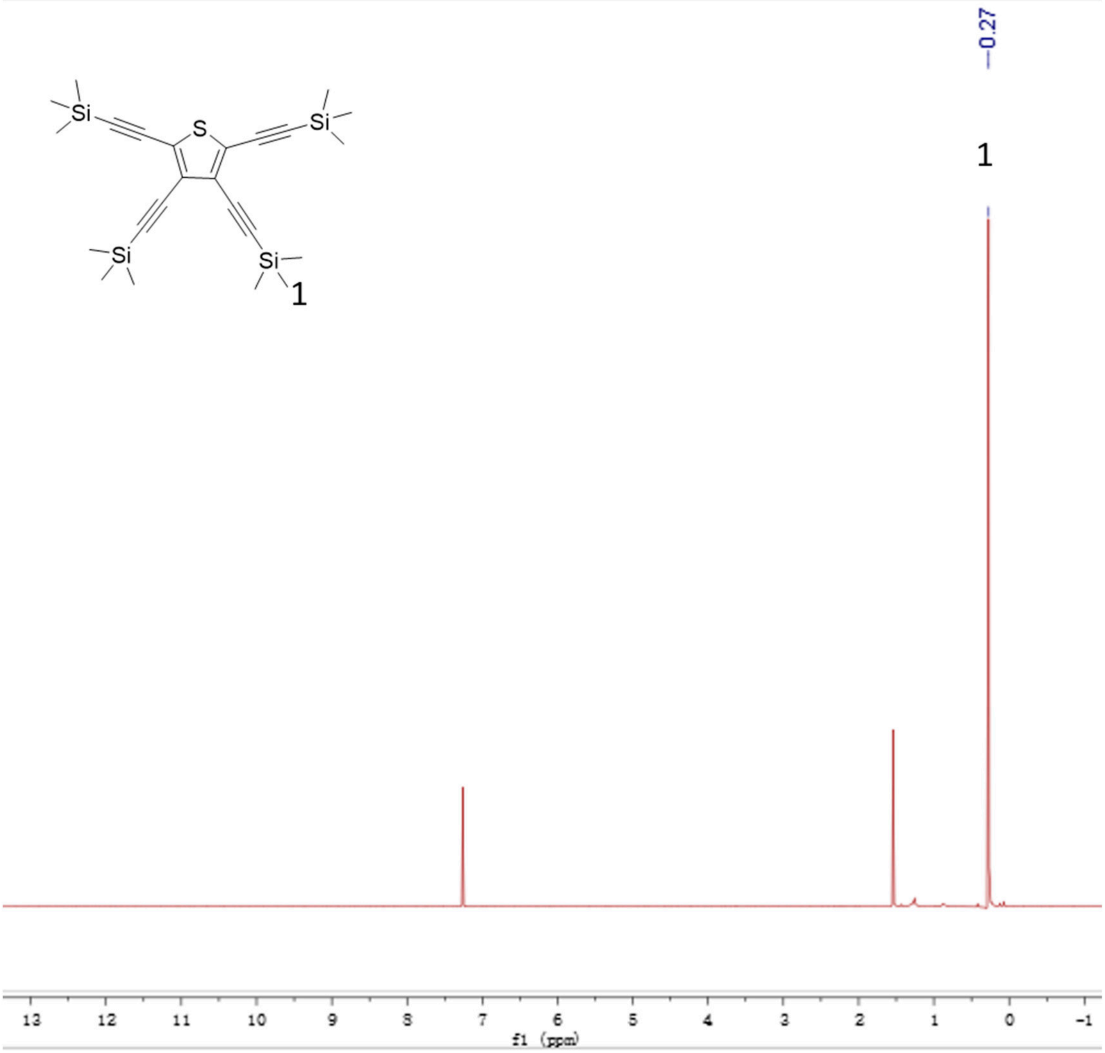

Figure S2. ^{1}H NMR spectrum of 2,3,4,5-tetrakis[(trimethylsilyl)ethynyl]-thiophene (**2**).

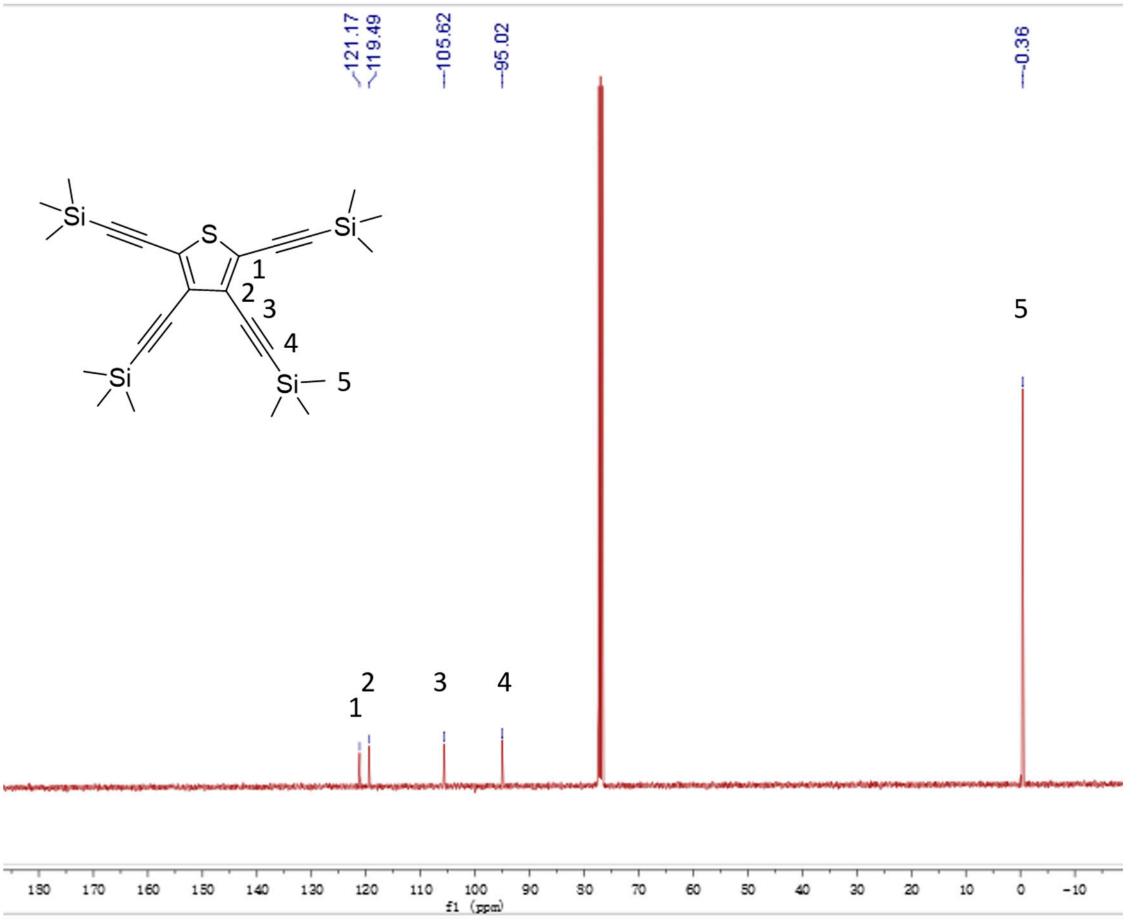

Figure S3. ^{13}C NMR spectrum of 2,3,4,5-tetrakis[(trimethylsilyl)ethynyl]-thiophene (**2**).

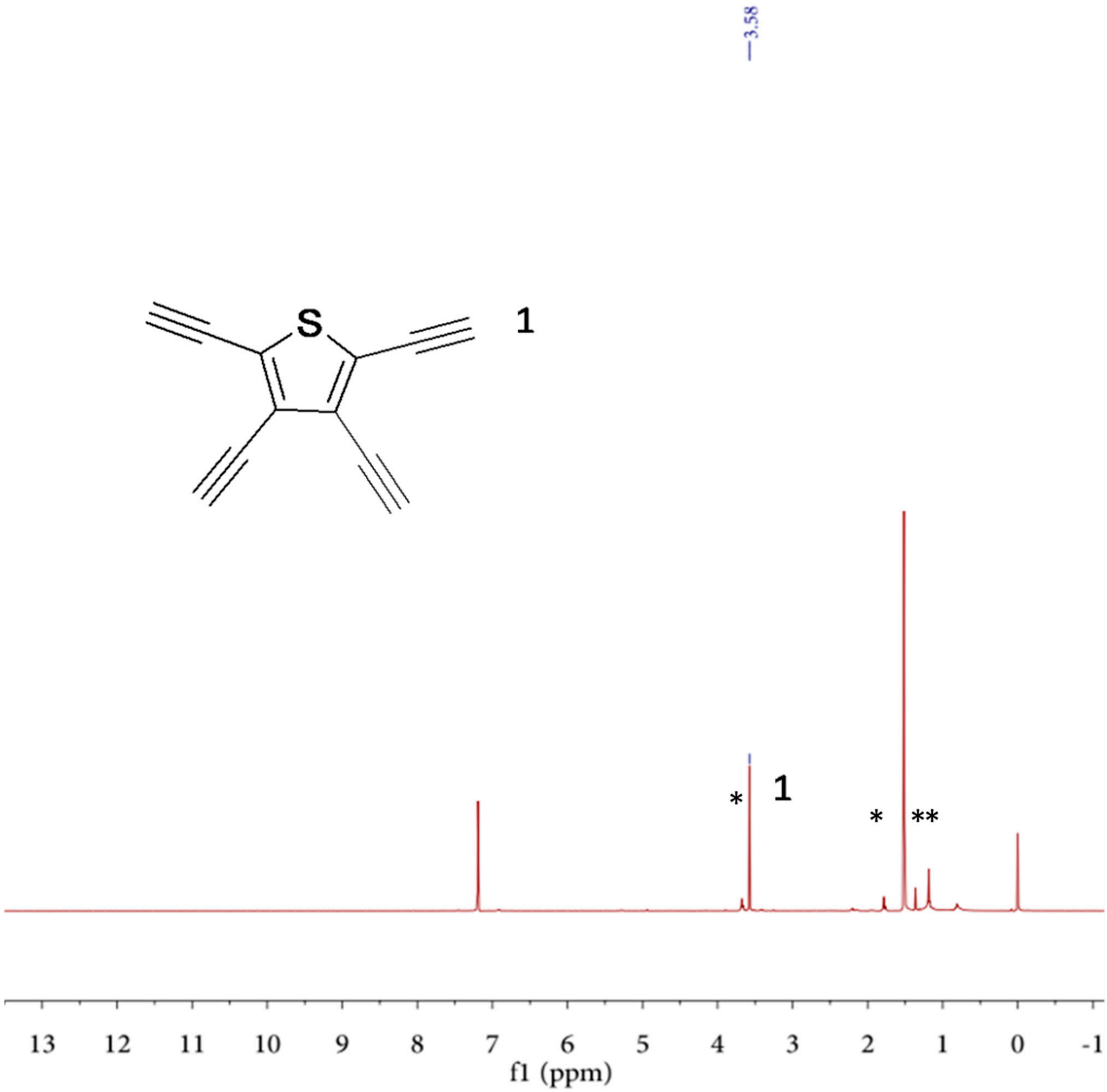

Figure S4. ^{1}H NMR spectrum of 2,3,4,5-tetraethynyl thiophene (**M1**). Attention: The NMR will be done immediately after the rotary evaporation since the monomer is unstable in the air. * may be caused by some residual solvent.

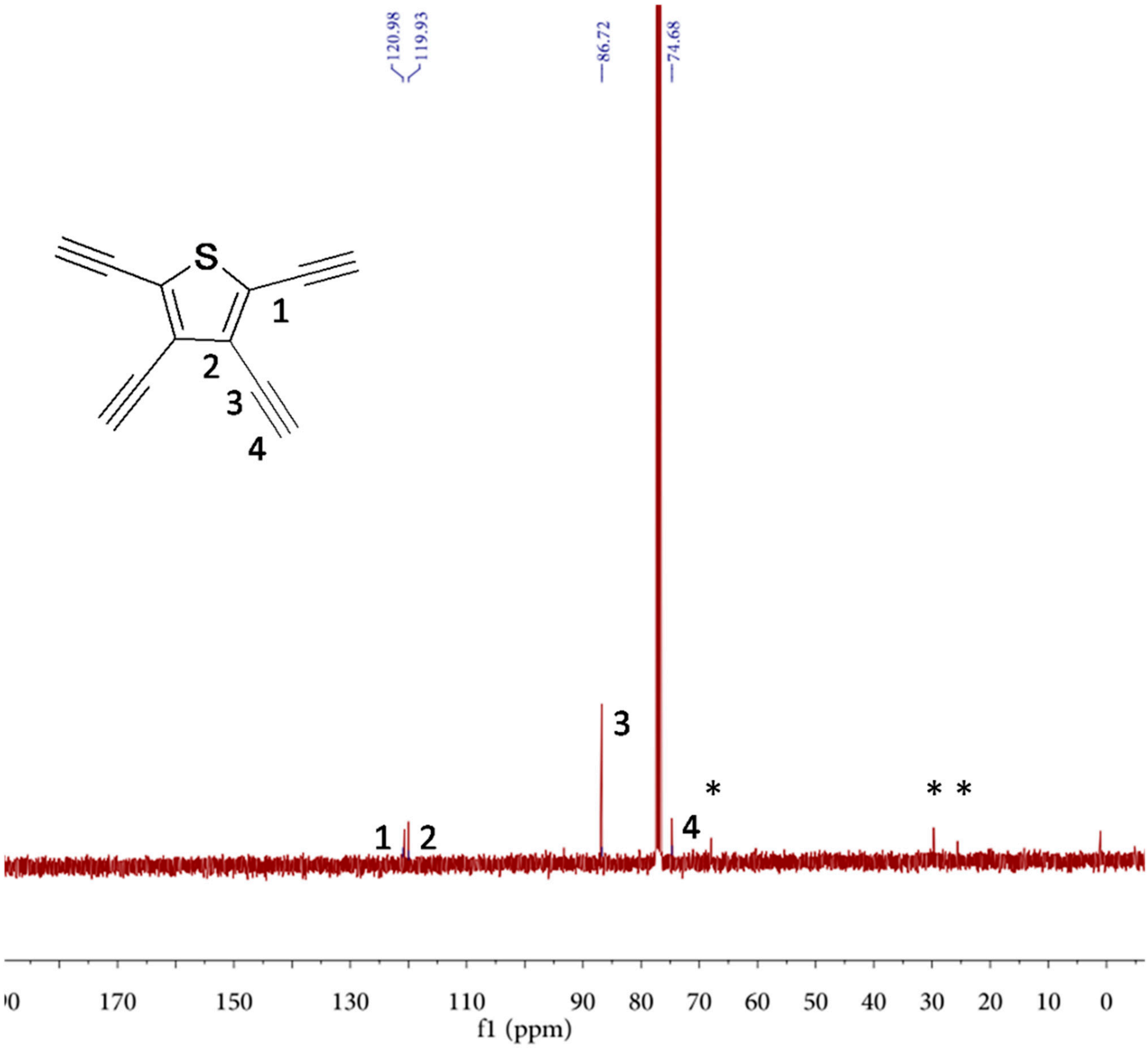

Figure S5. ^{13}C NMR spectrum of 2,3,4,5-tetraethynyl thiophene (**M1**). Attention: The NMR will be done immediately after the rotary evaporation since the monomer is unstable in the air. * may be caused by some residual solvent.

Section 2. Results of structure characterizations.

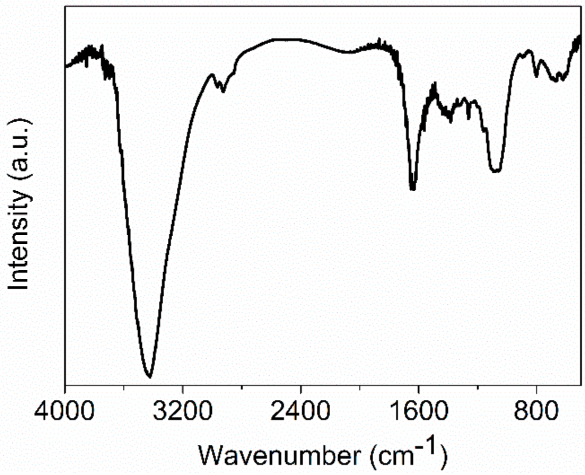

Figure S6. FT-IR spectrum of S-GDY.

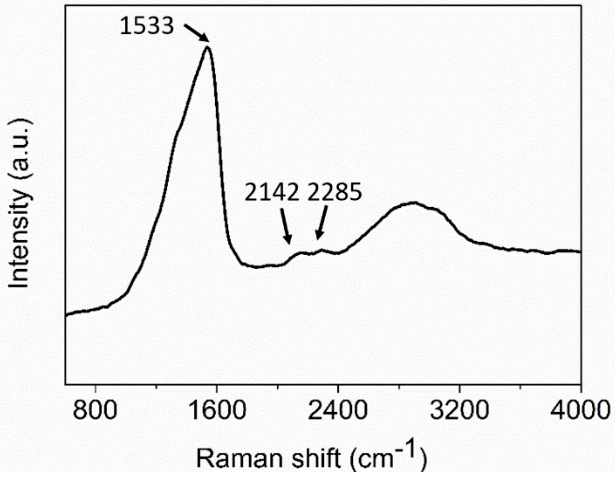

Figure S7. Raman spectrum of S-GDY.

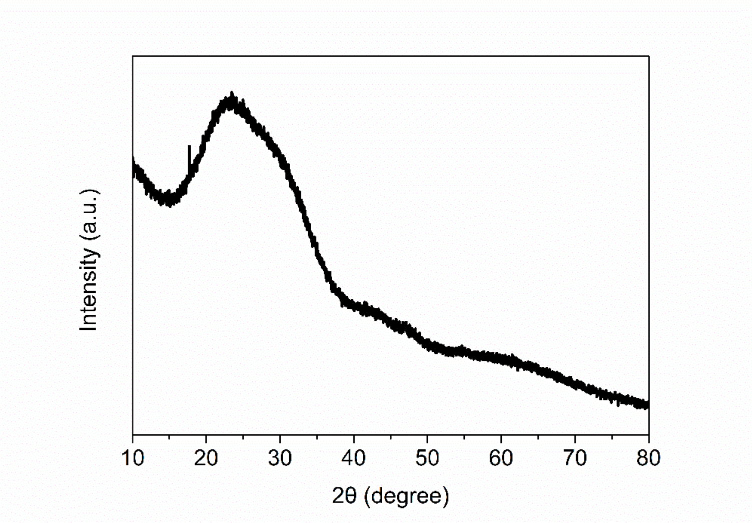

Figure S8. Power XRD pattern of S-GDY.

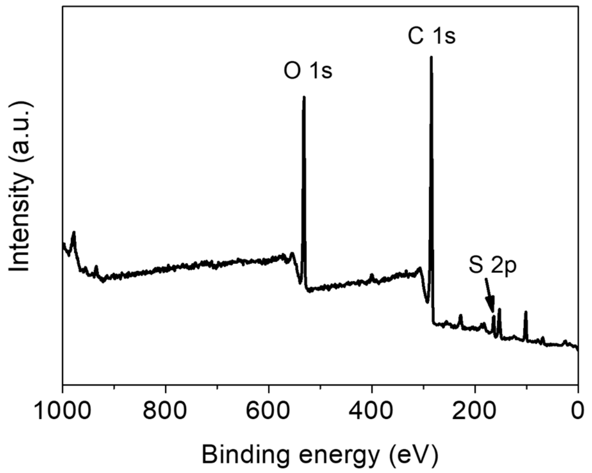

Figure S9. XPS survey spectrum of S-GDY.

Section 3. Results of porosity measurements.

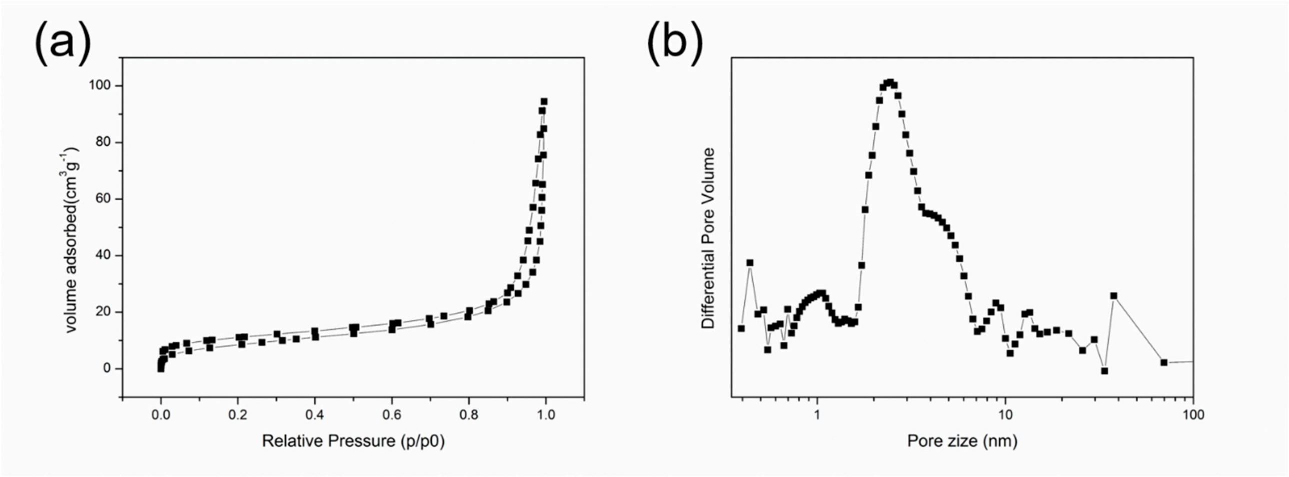

Figure S10. (**a**) Nitrogen adsorption-desorption isotherms of S-GDY, (**b**) pore size distribution profile from NLDFT calculation.

Section 4. Results of Electrochemical performances

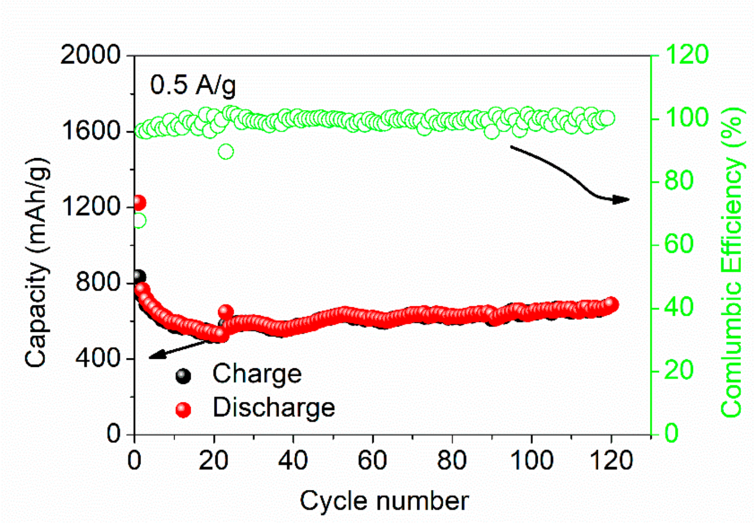

Figure S11. Long-term capacity retention at a current density of 0.5 A g^{-1}.

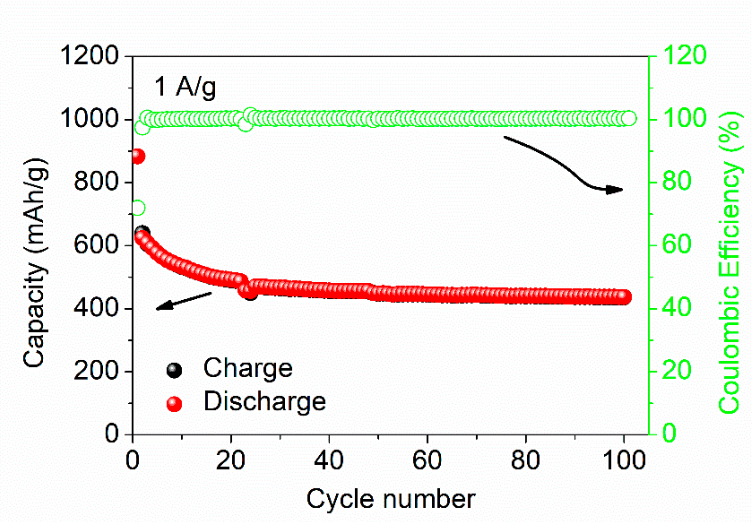

Figure S12. Long-term capacity retention at a current density of 1 A g^{-1}.

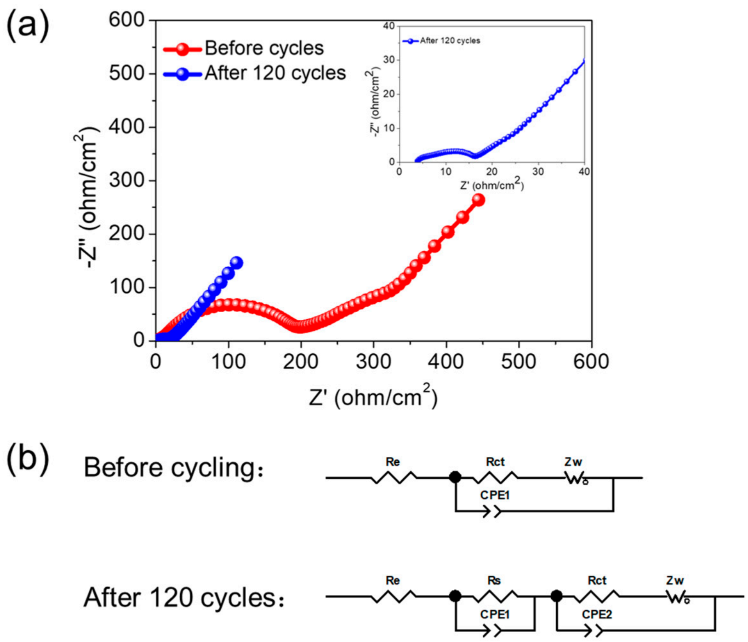

Figure S13. (**a**) Nyquist plots of the S-GDY electrode before and after 120 cycles at 0.5 A g^{-1}. The inset shows a S-GDY electrode after cycling alone in the enlarged part of the high-frequency region. (**b**) Equivalent circuits of the S-GDY electrode. In the equivalent circuit, R_e represents the electrolyte resistance; R_s is the equivalent resistance of the SEI layer formed on the electrode; R_{ct} is the charge-transfer resistance; Z_w is the Warburg impedance; CPE represents the corresponding double-layer capacitance.

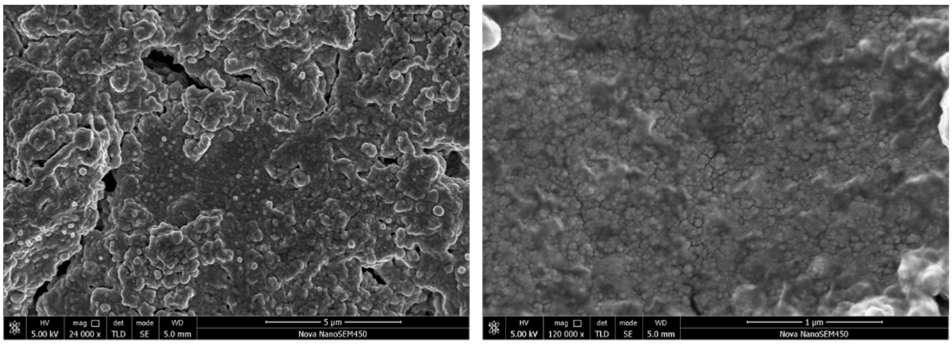

Figure S14. The SEM images of the S-GDY electrode after 120 cycles at 0.5 A g^{-1}.

Table S1. Comparison of performance of previously reported carbon-based materials.

Samples	Capacity (mA h g^{-1})	Current density (mA g^{-1})	Cycles
GDY film [1]	520	500	400
Bulk GDY [2]	552	50	200
N-GDY [3]	785	200	200
Activated carbon aerogels [4]	610	100	100
N-doped porous carbon materials [5]	488	100	100
Porous carbon [6]	833	100	50
NGY [7]	1037	100	200
S-GDY [8]	810	50	50
TTF-GDY [9]	837.6	50	200
This work	920	100	50

References

1. Huang, C.; Zhang, S.; Liu, H.; Li, Y.; Cui, G.; Li, Y. Graphdiyne for High Capacity and Long-Life Lithium Storage. *Nano Energy* **2015**, *11*, 481-489.
2. Zhang, S.; Liu, H.; Huang, C.; Cui, G.; Li, Y. Bulk Graphdiyne Powder Applied for Highly Efficient Lithium Storage. *Chem. Commun*. **2015**, *51*, 1834-1837.
3. Zhang, S.; Du, H.; He, J.; Huang, C.; Liu, H.; Cui, G.; Li, Y. Nitrogen-Doped Graphdiyne Applied for Lithium-Ion Storage. *ACS Appl. Mater. Interfaces* **2016**, *8*, 8467-8473.
4. Yang, X.; Wei, C.; Zhang, G. Activated Carbon Aerogels with Developed Mesoporosity as High-Rate Anodes in Lithium-Ion Batteries. *J. Mater. Sci.* **2016**, *51*, 5565-5571.
5. Zhang, X.; Zhu, G.; Wang, M.; Li, J.; Lu, T.; Pan, L. Covalent-Organic-Frameworks Derived N-Doped Porous Carbon Materials as Anode for Superior Long-Life Cycling Lithium and Sodium Ion Batteries. *Carbon* **2017**, *116*, 686-694.
6. Zhang, C.; Kong, R.; Wang, X.; Xu, Y.; Wang, F.; Ren, W.; Wang, Y.; Su, F.; Jiang, J.-X. Porous Carbons Derived from Hypercrosslinked Porous Polymers for Gas Adsorption and Energy Storage. *Carbon* **2017**, *114*, 608-618.
7. Yang, C.; Qiao, C.; Chen, Y.; Zhao, X.; Wu, L.; Li, Y.; Jia, Y.; Wang, S.; Cui, X. Nitrogen Doped gamma-Graphyne: A Novel Anode for High-Capacity Rechargeable Alkali-Ion Batteries. *Small* **2020**, *16*, 1907365.
8. Yang, Z.; Cui, W.; Wang, K.; Song, Y.; Zhao, F.; Wang, N.; Long, Y.; Wang, H.; Huang, C. Chemical Modification of the sp-Hybridized Carbon Atoms of Graphdiyne by Using Organic Sulfur. *Chem. - Eur. J*. **2019**, *25*, 5643-5647.
9. Pan, Q.; Chen, S.; Wu, C.; Zhang, Z.; Li, Z.; Zhao, Y. Sulfur-rich Graphdiyne-Containing Electrochemical Active Tetrathiafulvalene for Highly Efficient Lithium Storage Application. *ACS Appl. Mater. Interfaces* **2019**, *11*, 46070-46076.
